# Polymicrobial Catheter-Related Bloodstream Infection Involving *Brevundimonas diminuta* and *Streptococcus anginosus*: A Case Report and Literature Review

**DOI:** 10.3390/microorganisms14091888

**Published:** 2026-08-25

**Authors:** Po-Hsiu Huang, Chien-Hao Tseng, Chia-Wei Liu, Wei-Hsuan Huang, Ting-Kuang Yeh, Po-Yu Liu, Hsien-Po Huang

**Affiliations:** 1 Division of Infectious Diseases, Department of Internal Medicine, Taichung Veterans General Hospital, Taichung 407, Taiwan; 2 School of Medicine, College of Medicine, National Yang Ming Chiao Tung University, Taipei 112, Taiwan; 3 Institute of Molecular Biology, National Chung Hsing University, Taichung 402, Taiwan; 4 Graduate Institute of Biomedical Engineering, National Chung Hsing University, Taichung 402, Taiwan; 5 Institute of Clinical Medicine, National Yang Ming Chiao Tung University, Taipei 112, Taiwan; 6 Infection Control Center, Taichung Veterans General Hospital, Taichung 407, Taiwan; 7 Department of Post-Baccalaureate Medicine, College of Medicine, National Chung Hsing University, Taichung 402, Taiwan; 8 Doctoral Program in Translational Medicine, Rong Hsing Translational Medicine Research Center, National Chung Hsing University, Taichung 402, Taiwan

**Keywords:** *Brevundimonas diminuta*, *Streptococcus anginosus*, catheter-related bloodstream infection, co-infection

## Abstract

Catheter-related bloodstream infections (CRBSIs) caused by *Brevundimonas diminuta* are rare, and polymicrobial infections involving this organism are exceptionally uncommon. To the best of our knowledge, we describe the first reported case of polymicrobial CRBSI caused by *B. diminuta* and *Streptococcus anginosus* in a woman with a pituitary gland tumor and intracranial hypertension who was receiving intravenous treatment through an indwelling peripherally inserted central catheter (PICC). The patient made a full clinical recovery after prompt PICC removal and intravenous piperacillin–tazobactam therapy. This case emphasizes the emerging clinical significance of *B. diminuta* and *S. anginosus* as potential causes of polymicrobial bloodstream infection in patients with indwelling central venous catheters and underlines the need to consider uncommon environmental Gram-negative organisms in catheter-related infection.

## 1. Introduction

Catheter-related bloodstream infections are most commonly caused by skin commensals, particularly *Staphylococcus aureus* and *Staphylococcus epidermidis* [1]. By contrast, environmental bacteria are rarely implicated as causative pathogens in catheter-related bloodstream infections.

*Brevundimonas diminuta* is an aerobic, non-fermenting, Gram-negative bacillus commonly found in environmental reservoirs, including soil, water, and healthcare-associated settings. Although it has historically been regarded as a non-pathogenic organism, *B. diminuta* has increasingly been recognized as an opportunistic pathogen, particularly among immunocompromised and critically ill patients [2]. *Streptococcus anginosus* is a Gram-positive bacterium that commonly colonizes the upper respiratory, gastrointestinal, and reproductive tracts. Although it may remain as part of the normal flora, *S. anginosus* can cause localized noninvasive infections and may lead to invasive disease when it translocates into normally sterile body sites, such as the bloodstream or serosal cavities. Once invasive infection occurs, it can involve various tissues and organ systems throughout the body [3].

To our knowledge, although pyogenic liver abscess caused by co-infection with *B. diminuta* and *S. anginosus* has been reported in a patient with type 1 diabetes mellitus and chronic lymphocytic leukemia [4], catheter-related bloodstream co-infection involving both organisms has not previously been described. We therefore describe the first documented case of polymicrobial catheter-related bloodstream infection involving *B. diminuta* and *S. anginosus*, with emphasis on its diagnostic and clinical significance.

## 2. Case Presentation and Microbiological Findings

A 27-year-old woman with a non-functioning pituitary macroadenoma underwent transsphenoidal surgery without subsequent chemotherapy or radiotherapy. Following surgery, she received long-term cortisone therapy at a dose of 25 mg daily for six years. She presented to the emergency department with a three-day history of progressively worsening headache. She was admitted for management of intracranial hypertension, and intravenous therapy was administered through a single-lumen peripherally inserted central catheter (PICC). Dexamethasone 10 mg daily was also administered for three days to manage intracranial hypertension. On hospital day 15, she developed fever. Physical examination revealed a blood pressure of 114/64 mmHg, body temperature of 40.0 °C, pulse rate of 141 beats/min, and respiratory rate of 20 breaths/min. Examination of the head, eyes, ears, nose, and throat was unremarkable. There was no clubbing, cyanosis, lymphadenopathy, or icterus. Lung auscultation was clear, and no cardiac murmurs were detected. Bowel sounds were present, and there was no abdominal tenderness. Initial laboratory tests were notable for leukocytosis with neutrophil predominance, with a white blood cell count of 27,120/mm^3^ and neutrophils accounting for 88.2%, normocytic normochromic anemia with a hemoglobin level of 8.2 g/dL, and an elevated C-reactive protein level of 1.7 mg/dL. Her absolute neutrophil count was 23,920/mm^3^, and her serum cortisol level was 19.4 μg/dL. Cerebrospinal fluid analysis revealed no significant pleocytosis, with a total nucleated cell count of 2 cells/μL. Chest radiography showed no active pulmonary lesions, and abdominal computed tomography revealed no evidence of intra-abdominal infection. Urinalysis showed no pyuria, and the urine culture was negative. Oral and dental examinations were unremarkable. No alternative focus of infection was identified. No other documented causes of impaired immunity were identified, and she was not receiving any other immunosuppressive agents. Serum creatinine was 0.6 mg/dL with an estimated glomerular filtration rate of 119 mL/min/1.73 m^2^; renal function was normal and no dose adjustment was required. Empirical piperacillin–tazobactam 4.5 g every 6 h was started at the time of blood culture collection. Each dose was given as an extended 4 h infusion.

Blood cultures were drawn simultaneously from a peripheral vein and from the PICC at the onset of fever on hospital day 15, before the administration of antimicrobial agents. Both sets flagged positive within 24 h, and final identification with antimicrobial susceptibility results was available on hospital day 18. Isolates recovered from the peripheral vein and from the PICC-drawn blood cultures were subcultured separately and identified independently. Blood cultures obtained from both peripheral blood and the PICC grew *Brevundimonas diminuta* and *Streptococcus anginosus* (Supplementary Table S1). Both organisms were identified using the VITEK^®^ MS system with the IVD Knowledge Base version 3.2 (bioMérieux, Marcy-l’Étoile, France). *B*. *diminuta* was identified with a confidence value of 99.9% and *S*. *anginosus* with a confidence value of 99.9%, both above the manufacturer’s threshold for acceptable species-level identification. Antimicrobial susceptibility testing was performed using the VITEK^®^ 2 system (bioMérieux), software version 9.04. Testing was performed with the AST-N334 card for *B. diminuta* and the AST-ST03 card for *S. anginosus*. Results were interpreted according to Clinical and Laboratory Standards Institute (CLSI) M100, 34th edition (2024) [5]. We acknowledge that no species-specific breakpoints exist for *B. diminuta*. Categorical results were derived from the criteria for other non-Enterobacterales; for *S. anginosus*, the criteria for viridans group streptococci were applied. The *B. diminuta* isolate was susceptible to gentamicin, amikacin, trimethoprim-sulfamethoxazole, imipenem, ceftriaxone, cefepime, and piperacillin–tazobactam, but resistant to ceftazidime and ciprofloxacin. The *S. anginosus* isolate was susceptible to penicillin, vancomycin, ceftriaxone, and linezolid, but resistant to clindamycin and erythromycin. The same organisms with comparable antimicrobial susceptibility profiles were isolated from both peripheral and PICC-drawn blood cultures, and the detailed minimum inhibitory concentration (MIC) values are provided in Supplementary Table S2.

Catheter-related bloodstream infection was diagnosed based on accepted clinical and microbiological criteria, including isolation of the same organisms with identical susceptibility profiles from paired peripheral and catheter-drawn blood cultures, in the absence of another identifiable infectious focus. In this case, the PICC-drawn blood cultures became positive two hours earlier than the peripheral blood cultures, fulfilling the differential time to positivity (DTP) criterion for catheter-related bloodstream infection [6]. These findings supported the PICC as the most likely source of bloodstream infection, and the PICC was subsequently removed. Both aerobic blood culture bottles, one drawn from the PICC and the other from a peripheral vein, grew *B. diminuta* and *S. anginosus*. No microorganisms were recovered from the anaerobic bottles. Piperacillin–tazobactam 4.5 g every 6 h was continued after identification and susceptibility results became available. Two sets of follow-up blood cultures were drawn from peripheral veins 72 h after catheter removal; all bottles remained negative after 7 days of incubation. The patient completed 14 days of intravenous antimicrobial therapy, calculated from the initiation of appropriate therapy, and was subsequently discharged. The patient recovered fully after successful treatment with catheter removal and piperacillin–tazobactam, and was followed for 3 months after discharge, with no clinical or microbiological evidence of relapse. The patient’s chronological timeline is summarized in Table 1.

## 3. Discussion

*Brevundimonas diminuta* (formerly classified within the genus *Pseudomonas* and reassigned to the genus *Brevundimonas* by Segers et al. in 1994 [7]) is an aerobic, non-fermenting, Gram-negative bacillus belonging to the family Caulobacteraceae within the class Alphaproteobacteria. It is widely distributed in environmental reservoirs such as soil, water, and healthcare-associated settings, and has historically been regarded as an organism of low virulence [2]. In recent years, however, *B. diminuta* has been increasingly recognized as an emerging opportunistic pathogen, particularly among immunocompromised and critically ill patients. The broader adoption of modern identification platforms, most notably matrix-assisted laser desorption/ionization–time-of-flight mass spectrometry (MALDI-TOF MS), has facilitated its more reliable recognition by enabling accurate differentiation from other non-fermenting Gram-negative bacilli [2]. Although infections caused by *B. diminuta* remain uncommon, the organism is capable of producing severe, occasionally life-threatening disease, and its pathogenic potential in humans warrants continued investigation.

We searched PubMed/MEDLINE, Embase, Web of Science and Scopus from inception to 31 July 2026, without language restriction, combining the terms (‘*Brevundimonas diminuta*’ OR ‘*Pseudomonas diminuta*’) AND (catheter OR ‘central venous catheter’ OR ‘peripherally inserted central catheter’ OR bacteraemia OR bacteremia OR ‘bloodstream infection’ OR CRBSI OR CLABSI OR polymicrobial OR co-infection OR coinfection) AND (‘*Streptococcus anginosus*’ OR ‘*Streptococcus milleri*’ OR ‘*Streptococcus anginosus* group’). To the best of our knowledge, and on the basis of the literature search described above, this is the first reported case of polymicrobial catheter-related bloodstream infection involving *B. diminuta* and *S. anginosus* identified by our search. As an environmental organism, *B. diminuta* may also act as a co-pathogen in polymicrobial infections in immunocompromised hosts, and Table 2 summarizes previously reported cases of *B. diminuta* co-infection. In the largest series to date, Han and Andrade [8] described eight *B. diminuta* isolates recovered from seven patients, all of whom had an underlying malignancy. Four patients had co-infections involving other pathogens, including coagulase-negative *Staphylococcus*, *Moraxella osloensis*, *Enterococcus* species, and *Staphylococcus aureus*. The isolates were recovered from bloodstream, intravascular catheter, urinary tract, and pleural specimens. Burch et al. [4] described a 67-year-old man with type 1 diabetes mellitus and chronic lymphocytic leukemia in whom *B. diminuta* and *S. anginosus* were co-isolated from a pyogenic liver abscess; the infection resolved after drainage and meropenem therapy. More recently, Chen et al. [9] reported meningitis caused by co-infection with *Lysinibacillus sphaericus* and *B. diminuta* in a 10-year-old boy with cerebral atrophy, which resolved with meropenem and gentamicin. Consistent with our case, most reported *B. diminuta* infections have been associated with immunosuppression, malignancy, or indwelling vascular devices.

A review of the literature (Table 3) shows that *B. diminuta* catheter-related bloodstream infection most frequently affects patients with hematologic malignancy, with favorable outcomes once appropriate antimicrobial therapy is administered and, where feasible, the catheter is removed. In earlier cases, the catheter was often retained [8], whereas more recent reports have favored prompt device removal [11]. From a clinical standpoint, *S. anginosus*, a member of the *Streptococcus anginosus* group, is a commensal of the oral, gastrointestinal, and genitourinary tracts that is nonetheless distinguished by a marked propensity for abscess formation and invasive pyogenic disease once it reaches normally sterile sites [3]. The group is likewise recognized for its capacity to participate in biofilm-associated and polymicrobial infections, in which synergistic interactions with co-infecting organisms may enhance tissue invasion and persistence [3]. In the setting of co-infection, the presence of *S. anginosus* may therefore contribute to biofilm persistence on the indwelling catheter and to greater therapeutic complexity, further underscoring the importance of timely catheter removal and appropriate antimicrobial selection.

Compared with previously reported cases, the coexistence of *B. diminuta* and *S. anginosus* has, to our knowledge, been described only once before. Burch et al. [4] reported a pyogenic liver abscess caused by co-infection with these two organisms in a patient with type 1 diabetes mellitus and chronic lymphocytic leukemia, in whom the two species were presumed to act synergistically within the abscess cavity; however, no intravascular device was implicated as the source. To the best of our knowledge, our report is therefore the first documented catheter-related bloodstream co-infection involving both pathogens. The mechanisms underlying such co-infection remain speculative: the coexistence of an environmental Gram-negative bacillus and a pyogenic streptococcus may reflect biofilm synergy on the indwelling catheter or transient translocation from colonized skin or mucosal surfaces, and merits further investigation, particularly in immunocompromised hosts or patients with indwelling intravascular access.

Antimicrobial susceptibility testing in our case revealed that the *B. diminuta* isolate was susceptible to gentamicin, amikacin, trimethoprim-sulfamethoxazole, imipenem, ceftriaxone, cefepime, and piperacillin–tazobactam, but resistant to ceftazidime and ciprofloxacin. The fluoroquinolone resistance observed in our isolate is consistent with the findings of Han and Andrade [8], who identified amino acid substitutions in the fluoroquinolone target proteins as the probable basis of resistance in *B. diminuta*. A substantial contribution from efflux-mediated mechanisms was not demonstrated in that study and remains speculative. Although the organism generally remains susceptible to carbapenems and aminoglycosides, sporadic reports of VIM-2 metallo-β-lactamase-producing and colistin-resistant *B. diminuta* indicate the potential for emerging and even extensively drug-resistant phenotypes [12,13]. The co-infecting *S. anginosus* isolate was susceptible to penicillin, vancomycin, ceftriaxone, and linezolid, but resistant to clindamycin and erythromycin, a pattern in keeping with the macrolide and lincosamide resistance frequently observed within this group [14]. Notably, because *B. diminuta* is commonly resistant to fluoroquinolones, empirical fluoroquinolone monotherapy would have been ineffective in this case; the favorable response to piperacillin–tazobactam therefore highlights the importance of susceptibility-guided therapy for this organism. Continuous surveillance and molecular characterization of resistance determinants are accordingly warranted, particularly in nosocomial settings.

Given the ubiquity of *B. diminuta* in water, soil, and healthcare environments, environmental exposure, including contact with contaminated water, dust, or flushing solutions, could have contributed to colonization or contamination of the vascular access device [2]. Although direct environmental cultures were not obtained, the clinical course and microbiological findings were consistent with a catheter-related source of infection. The successful management of this case with piperacillin–tazobactam and prompt removal of the peripherally inserted central catheter reinforces the importance of combining appropriate antimicrobial therapy with elimination of the infected device. As observed in prior reports, catheter removal remains decisive for environmental Gram-negative catheter-related bloodstream infection, since persistent biofilm colonization may otherwise lead to relapse despite in vitro susceptibility. This case report has several limitations. Species identification relied solely on the VITEK^®^ MS system without confirmation by 16S rRNA gene sequencing or whole-genome sequencing. Therefore, the species-level identification should be interpreted with caution, as the VITEK^®^ MS system may not reliably distinguish between closely related species. In addition, the catheter tip was not sent for culture; the diagnosis therefore rests on the differential time to positivity criterion.

## 4. Conclusions

This case underscores the clinical significance of *Brevundimonas diminuta* and *Streptococcus anginosus* as potential pathogens involved in polymicrobial catheter-related bloodstream infection. Clinicians should remain alert to the possibility of uncommon organisms when assessing bloodstream infections in patients with indwelling vascular catheters. Further genomic and environmental studies are warranted to clarify the reservoirs, virulence determinants, and potential interactions of *B. diminuta* with *Streptococcus* species.

## Figures and Tables

**Table 1:** Chronological timeline of the patient.

Event	Time
Admission and PICC insertion	Hospital day 1
Onset of fever	Hospital day 15
Paired blood cultures collected	Hospital day 15
Empirical piperacillin–tazobactam started	Hospital day 15
Blood cultures flagged positive (PICC/peripheral)	Hospital day 16
PICC removed	Hospital day 16
Final identification and susceptibility available	Hospital day 18
Follow-up blood cultures collected	Hospital day 19
Final confirmation of negative follow-up cultures	Hospital day 26
Completion of 14-day antimicrobial therapy	Hospital day 28
Discharge	Hospital day 30
Outpatient follow-up (no relapse)	3 months after discharge

Abbreviation: PICC, peripherally inserted central catheter.

**Table 2:** Clinical characteristics of patients with *Brevundimonas diminuta* co-infection.

Year/ Country	Age/ Gender	Underlying Diseases	Co-Infection Pathogen	Site/Source	Treatment	Outcome	Reference
2005, USA	26, Male	Leukemia	Coagulase-negative *Staphylococcus*	CRBSI	VAN and FEP	Clinical resolution	[8]
	54, Male	Leukemia, bone marrow transplantation	*Moraxella osloensis*	CRBSI	LVX	Clinical resolution	
	55, Male	Sarcoma	*Enterococcus* sp.	UTI	LVX	Clinical resolution	
	41, Male	Sarcoma	*Staphylococcus aureu* *s*	Empyema	TIM	Clinical resolution	
2011, Taiwan (n = 2) *	N/A	Hematologic malignancy	*Agrobacterium radiobacter* and coagulase-negative *Staphylococcus*	Bacteremia	N/A	N/A	[10]
2021, USA	67, Male	Type 1 diabetes mellitus, chronic lymphocytic leukemia	*Streptococcus* *anginosus*	Pyogenic liver abscess	MEM	Clinical resolution	[4]
2026, China	10, Male	Cerebral atrophy	*Lysinibacillus sphaericus*	Meningitis	MEM and GEN	Clinical resolution	[9]
2026, Taiwan (This case)	27, Female	Pituitary gland tumor	*Streptococcus* *anginosus*	CRBSI	TZP	Clinical resolution	

* Individual-level age, gender, treatment, and outcome data were unavailable. Abbreviations: N/A, not available; CRBSI, catheter-related bloodstream infection; UTI, urinary tract infection; VAN, vancomycin; FEP, cefepime; LVX, levofloxacin; TIM, ticarcillin/clavulanate; MEM, meropenem; GEN, gentamicin; TZP, piperacillin–tazobactam.

**Table 3:** Clinical characteristics of patients with catheter-related bloodstream infections caused by *Brevundimonas diminuta*.

Year/ Country	Age/ Gender	Underlying Diseases	CRBSI Type *	Antibiotic Susceptibility	Antibiotic Resistance	Treatment	Outcome	Catheter Removal	Reference
2005, USA	26, Male	Leukemia	Reported	AMK, IPM, TIM, SXT, CRO	CAZ, FEP, AMP, GAT, LVX, CIP	VAN and FEP	Clinical resolution	No	[8]
	69, Male	Lymphoma	Reported	AMK, IPM, TIM, SXT	CRO, CAZ, FEP, AMP, GAT, LVX, CIP	IPM, NAF and LVX	Clinical resolution	No	
	74, Male	Leukemia	Reported	AMK, IPM, TIM	SXT, CAZ, AMP, CIP	MEM and TOB	Clinical resolution	No	
	37, Female	Lymphoma, bone marrow transplantation	Reported	AMK, IPM, TIM, CAZ	SXT, AMP, CIP	TIM and NAF	Clinical resolution	No	
	54, Male	Leukemia, bone marrow transplantation	Reported	AMK, IPM, TIM	SXT, CRO, CAZ, FEP, AMP, GAT, LVX, CIP	LVX	Clinical resolution	No	
2026, Morocco	18, Female	Hodgkin lymphoma	Suspected	MEM, IPM, AMK, TOB, PIP, TIC, TZP	CAZ, ATM, LVX, CIP	IPM	Clinical resolution	Yes	[11]
2026, Taiwan (This case)	27, Female	Pituitary gland tumor	Definitive	GEN, AMK, SXT, IPM, CRO, FEP, TZP	CAZ, CIP	TZP	Clinical resolution	Yes	

* Definitive CRBSI: The same organism is isolated from a catheter-tip culture and from at least one percutaneous blood culture; or paired blood cultures (catheter and peripheral) fulfil the quantitative blood culture or DTP criteria [6]. Reported CRBSI: Although blood cultures obtained from the central line were positive and the authors of the previous case report considered the case to represent CRBSI, the available evidence does not appear to fully satisfy the 2009 IDSA diagnostic criteria for CRBSI. Suspected CRBSI: Blood cultures obtained from peripheral blood were positive, and the most likely source of infection was a catheter. Abbreviations: CRBSI, catheter-related bloodstream infection; VAN, vancomycin; AMK, amikacin; IPM, imipenem; NAF, nafcillin; TIM, ticarcillin/clavulanate; SXT, trimethoprim-sulfamethoxazole; CRO, ceftriaxone; CAZ, ceftazidime; FEP, cefepime; AMP, ampicillin; GAT, gatifloxacin; LVX, levofloxacin; CIP, ciprofloxacin; MEM, meropenem; TOB, tobramycin; PIP, piperacillin; TIC, ticarcillin; ATM, aztreonam; GEN, gentamicin; TZP, piperacillin–tazobactam.

## Data Availability

The original contributions presented in this study are included in the article/Supplementary Materials. Further inquiries can be directed to the corresponding author.

## Supplementary Materials

The following supporting information can be downloaded at: https://www.mdpi.com/article/10.3390/microorganisms14091888/s1, Table S1: Number of sets and bottles, organisms recovered, and time to positivity; Table S2: Minimum Inhibitory Concentrations of the isolates.
