# Supplementary material for: Polymicrobial Catheter-Related Bloodstream Infection Involving Brevundimonas diminuta and Streptococcus anginosus: A Case Report and Literature Review
Source: Microorganisms. 2026 Aug 25;14(9):1888. doi: 10.3390/microorganisms14091888 (PMC13609207; doi:10.3390/microorganisms14091888)
Supplement: Supplementary file 1 [file microorganisms-14-01888-s001.zip › microorganisms-4479618-supplementary.pdf]

Supplementary Table S1. Number of sets and bottles, organisms recovered, and time to positivity.

Sampling site	Set number	Bottle	Organisms recovered	Time to positivity
PICC	1	Aerobic	*Brevundimonas diminuta* and *Streptococcus anginosus*	16 hours
PICC	1	Anaerobic	No organisms recovered	-
Peripheral vein	2	Aerobic	*Brevundimonas diminuta* and *Streptococcus anginosus*	18 hours
Peripheral vein	2	Anaerobic	No organisms recovered	-

Abbreviation: PICC, peripherally inserted central catheter.

Supplementary Table S2. Minimum Inhibitory Concentrations of the isolates.

Organism	Antimicrobial agent	MIC (μg/mL), peripheral isolate	MIC (μg/mL), PICC isolate
Brevundimonas diminuta	Gentamicin	4 (S)	4 (S)
	Amikacin	≤ 2 (S)	≤ 2 (S)
	TMP-SMX	2/38 (S)	2/38 (S)
	Imipenem	2 (S)	2 (S)
	Ceftazidime	32 (R)	32 (R)
	Cefepime	8 (S)	8 (S)
	Ceftriaxone	8 (S)	8 (S)
	Ciprofloxacin	4 (R)	4 (R)
	Piperacillin-tazobactam	≤ 4 (S)	≤ 4 (S)
Streptococcus anginosus	Clindamycin	≥ 1 (R)	≥ 1 (R)
	Penicillin	≤ 0.06 (S)	≤ 0.06 (S)
	Vancomycin	0.5 (S)	0.5 (S)
	Ceftriaxone	≤ 0.12 (S)	≤ 0.12 (S)
	Erythromycin	8 (R)	8 (R)
	Linezolid	≤ 2 (S)	≤ 2 (S)

Abbreviation: MIC, minimum inhibitory concentration; PICC, peripherally inserted central catheter; TMP-SMX, trimethoprim-sulfamethoxazole; S, susceptible; R, resistant.
